# Selective Budding of SARS-CoV-Like Particles from Glycolipid-Enriched Membrane Lipid Rafts and Host Gene Modulation

**DOI:** 10.3390/microorganisms14010159

**Published:** 2026-01-10

**Authors:** Manoj K. Pastey, Yue Huang, Barney Graham

**Affiliations:** 1 Department of Veterinary Biomedical Sciences, Oregon State University, Corvallis, OR 97330, USA; 2 Vaccine Research Center, National Institutes of Allergy and Infectious Diseases, National Institutes of Health, Bethesda, MD 20892, USA

**Keywords:** SARS-CoV, virus-like particles (VLPs), lipid rafts, cholesterol-rich microdomains, viral assembly, spike protein, coronavirus morphogenesis, host gene modulation, ERGIC/Golgi membranes

## Abstract

Severe acute respiratory syndrome coronavirus (SARS-CoV) assembles and buds from the Golgi apparatus or the ER membrane, but the specific membrane microdomains utilized during this process remain underexplored. Here, we show that co-expression of the SARS-CoV structural proteins S, M, and N in HEK-293T cells is sufficient to generate genome-free SARS-CoV-like virus-like particles (VLPs), which preferentially bud from glycolipid-enriched membrane lipid raft microdomains. Immunofluorescence microscopy using raft-selective dyes (DiIC16) and spike-specific antibodies revealed strong co-localization of VLPs with lipid rafts. Detergent-resistant membrane analysis and sucrose gradient centrifugation further confirmed the presence of S protein in buoyant, raft-associated fractions alongside the raft marker CD44. Importantly, pharmacological disruption of rafts with methyl-β-cyclodextrin reduced VLP budding and S protein partitioning into raft domains, underscoring the requirement for intact lipid rafts in assembly. Additionally, our data support lipid raft-associated proteins’ (e.g., FNRA, VIM, CD59, RHOA) roles in modulating cellular responses conducive to viral replication and assembly. These findings highlight lipid rafts as crucial platforms for SARS-CoV morphogenesis and suggest new avenues for vaccine and antiviral development using VLPs and raft-targeting therapeutics.

## 1. Introduction

Lipid rafts are dynamic, cholesterol- and sphingolipid-enriched microdomains of the cell membranes that act as platforms for signal transduction, protein sorting, and pathogen entry [1]. Increasing evidence indicates that many enveloped viruses, including HIV, influenza, respiratory syncytial virus (RSV), Ebola, and measles, hijack lipid rafts for their assembly and budding [2,3,4,5,6,7]. These domains concentrate viral proteins, facilitate glycoprotein clustering, and interact with host factors that coordinate membrane curvature and scission. As such, lipid rafts have emerged as strategic targets for antiviral drug development [8,9].

Severe acute respiratory syndrome coronavirus (SARS-CoV), a positive-sense RNA virus of the Coronaviridae family, was responsible for the 2002–2003 SARS outbreak [10] and again in 2019–2021 (COVID-19), which was caused by the SARS-CoV-2 [11]. Like other coronaviruses, SARS-CoV encodes four structural proteins: spike (S), membrane (M), nucleocapsid (N), and envelope (E). While the E protein plays roles in virion stability and ion channel activity, the minimal components required for virion morphogenesis are S, M, and N [12]. The M protein orchestrates viral assembly by interacting with both S and N; S mediates host cell entry via receptor binding and membrane fusion, and N packages the viral genome into a ribonucleoprotein complex [12,13].

Recently, considerable attention has focused on the role of lipid rafts in the lifecycle of SARS-CoV, and lipid rafts have been suggested as significant factors in the infection of host cells [14]. Specifically, cholesterol- and sphingolipid rich lipid raft microdomains in the plasma membrane have been shown to be involved in SARS-CoV entry through an endocytic pathway that is independent of clathrin and caveolae but is dependent on rafts and involves viral attachment to angiotensin converting enzyme 2 (ACE2) in the host cell plasma membrane [14,15,16,17].

The selective use of lipid rafts by SARS-CoV during particle assembly remains poorly characterized. The effect of S-acylation on membrane lipid organization of SARS-CoV-2 and its infectivity [18] suggests that lipid rafts are also involved in virus assembly. S-palmitoylation of the spike protein not only results in the formation of viruses with enhanced fusion capacity, but also facilitates the formation of localized, ordered cholesterol and sphingolipid-rich lipid nanodomains in the early Golgi (or the intermediate compartment between the ER and the Golgi), where viral budding occurs, as well as generating cholesterol-rich lipid domains within viral envelopes [18]. It has been suggested that acyl moieties recruit cholesterol into close proximity to the spike protein, thus triggering the formation of ordered nanodomains [18]. Given the involvement of rafts in the life cycles of related enveloped viruses, we hypothesized that SARS-CoV structural proteins may similarly exploit these lipid raft-like microdomains from the Golgi apparatus or the ER for efficient budding. Understanding these interactions has implications for vaccine development—especially VLP-based platforms—and for antiviral strategies targeting membrane domains.

In this study, we explored the ability of SARS-CoV structural proteins to assemble into VLPs and examined whether these particles preferentially bud from lipid rafts. While prior studies have demonstrated the role of lipid rafts in viral entry, our data provide direct experimental evidence that structural SARS-CoV proteins alone are sufficient to induce VLP formation at raft domains, even in the absence of viral genome replication. We used fluorescence imaging, lipid dye labeling, detergent-insoluble membrane fractionation, and sucrose gradient analysis to dissect viral protein localization. Finally, we disrupted rafts with methyl-β-cyclodextrin to evaluate the dependence of budding on cholesterol-rich domains. Additionally, our data support lipid raft-associated proteins’ roles in modulating cellular responses conducive to viral replication and assembly. Our findings provide mechanistic insight into SARS-CoV morphogenesis and establish a framework for leveraging lipid rafts in antiviral and vaccine design.

## 2. Materials and Methods

### 2.1. Cell Culture

HEK-293T cells (ATCC CRL-3216) were maintained in Dulbecco’s Modified Eagle Medium (DMEM; Gibco, Grand Island, NY, USA) supplemented with 10% heat-inactivated fetal bovine serum (FBS), 2 mM L-glutamine, and 100 U/mL penicillin-streptomycin at 37 °C with 5% CO_2_. HEK-293T cells were chosen due to their high transfection efficiency and suitability for producing virus-like particles.

### 2.2. Synthetic SARS-CoV Expression Vectors and Transfection

Synthetic SARS-CoV expression vectors were constructed as previously described [12,19,20]. Briefly, protein sequences corresponding to the SARS-CoV Urbani strain structural proteins Spike (S), Nucleocapsid (N), Membrane (M), and Envelope (E), as documented in GenBank (accession number AY278741) [13], were reverse-translated into human cell-preferred codons using the Wisconsin Genetics Computer Group (GCG) software package (version 11.1; Accelrys Inc., San Diego, CA, USA accessed via https://www.accelrys.com on 21 June 2020). Subsequently, oligonucleotides covering these four genes were synthesized commercially by Sigma-Genosys, each oligonucleotide being 75 bases long with an overlap of 25 bases between consecutive segments. The codon-optimized genes were then assembled into expression vectors derived from the pNGVL-3 plasmid backbone and verified through sequencing to confirm accuracy and integrity. Plasmid DNA was purified by double cesium chloride gradient ultracentrifugation to ensure high purity suitable for transfection.

For transfection, HEK-293T cells were plated at a density of 3 × 10^6^ cells per well in six-well plates and transfected with 2 µg each of the plasmids encoding S, M, and N proteins (totaling 6 µg DNA per transfection) using the calcium phosphate precipitation method as described previously [21]. The vector backbone without viral inserts was used as filler DNA to maintain consistent total DNA amounts across experimental conditions.

### 2.3. Immunofluorescence and Lipid Dye Labeling

To investigate the association between SARS-CoV structural proteins and lipid raft microdomains, we employed immunofluorescence microscopy coupled with selective lipid raft labeling. DiIC_16_ (3) (1,1-dihexadecyl-3,3,3,3-tetramethylindocarbocyanine perchlorate) consists of a long acyl chain, which allows its incorporation into the more rigid, ordered lipid domains, compared to DiIC_12_ (3) (1,1-didodecyl-3,3,3,3-tetramethylindocarbocyanine perchlorate), which labels the fluid nonraft membrane components [22]. Briefly, at 48 h post-transfection, cells seeded on coverslips were chilled on ice and incubated with a 1:100 dilution of lipid raft-selective dye DiIC_16_ (3) or non-raft-selective dye DiIC_12_ (3) dissolved in ethanol for 15 min. Subsequently, cells were fixed with 3.7% formaldehyde for 10 min at room temperature, permeabilized with 0.5% Triton X-100 (VWR International, LLC, Radnor, PA, USA) in phosphate-buffered saline (PBS) for 10 min and blocked with 5% non-fat milk in PBS for 15 min to minimize nonspecific binding. Cells were then incubated for 1 h at room temperature with primary antibodies: either human anti-S monoclonal antibody or mouse anti-M monoclonal antibody (CDC), diluted at 1:500 in 3% milk-PBS. Following washing with PBS-Tween-20, cells were incubated for 1 h with Alexa Fluor 488-conjugated secondary antibodies (anti-human or anti-mouse IgG, Molecular Probes) diluted 1:1000 in 3% milk-PBS. Nuclear counterstaining was performed with DAPI. Images were captured using a Zeiss Axioplan fluorescence microscope (Carl Zeiss Microscopy GmbH, Jena, Germany) equipped with a 100× oil immersion objective, and digital images were acquired using an Axiocam camera (Carl Zeiss Microscopy GmbH, Jena, Germany).

### 2.4. Detergent Extraction and Sucrose Gradient Fractionation

To biochemically assess the association of SARS-CoV structural proteins with lipid raft microdomains, detergent-resistant membrane fractionation was performed using sucrose density gradients. Briefly, at 48 h post-transfection, cells were lysed in cold TNE buffer (50 mM Tris-HCl pH 7.5, 150 mM NaCl, 5 mM EDTA, 1% Triton X-100, and protease inhibitor cocktail) for 30 min at 4 °C. Lysates were clarified by centrifugation at 2000 rpm for 10 min at 4 °C to remove nuclei and debris. The post-nuclear supernatant was carefully layered beneath discontinuous sucrose gradients composed of 5%, 35%, and 80% sucrose in TNE buffer. Gradients were centrifuged at 35,000 rpm for 16 h at 4 °C in a Beckman SW41 rotor. Twelve 1 mL fractions were collected sequentially from the top to the bottom of the gradient for downstream analysis. Fractions 3 to 5 were designated as raft-enriched based on CD44 distribution, while fractions 7 to 11 represented non-raft regions marked by the transferrin receptor. Enrichment of spike protein in CD44-positive fractions further supports localization of viral assembly to lipid raft microdomains.

### 2.5. Protein Precipitation, Analysis, and Blotting

To evaluate the distribution of viral and host proteins within raft and non-raft membrane fractions, proteins were precipitated, resolved, and analyzed by Western blotting. Briefly, samples from the sucrose gradient fractions were subjected to methanol/chloroform precipitation as described previously [23]. Briefly, 200 µL aliquots were mixed with 0.8 mL methanol and 0.2 mL chloroform, vortexed, and centrifuged. To separate phases, 0.6 mL distilled water was added, followed by further vortexing and centrifugation at 13,000 rpm for 1 min. The upper aqueous phase was discarded, and proteins were re-precipitated with 0.6 mL methanol and centrifuged again at 13,000 rpm for 2 min. Pellets were resuspended in 5% sodium dodecyl sulfate (SDS), and protein concentration was determined using a BCA protein assay kit (Pierce, Rockford, IL, USA). Equal amounts of protein from fractions 3 to 11 were separated by SDS-polyacrylamide gel electrophoresis (SDS-PAGE) and transferred to membranes for Western blotting. Blots were probed with primary antibodies against the SARS-CoV spike protein (Imgenex Corporation, San Diego, CA, USA), CD44 (lipid raft marker; Sigma-Aldrich, St. Louis, MO, USA), and transferrin receptor (non-raft marker; Zymed Laboratories, South San Francisco, CA, USA). Horseradish peroxidase (HRP)-conjugated secondary antibodies were used for detection, and signals were visualized using enhanced chemiluminescence (ECL; Pierce).

### 2.6. Lipid Raft Disruption

To investigate the involvement of cholesterol-rich lipid rafts during the assembly steps of SARS-CoV infection, we used methyl-β-cyclodextrin to deplete cholesterol from HEK-293T cells. At 48 h post-transfection, cells were treated with 10 mM methyl-β-cyclodextrin (Sigma) for 30 min at 37 °C, followed by two PBS washes. Treated cells were then labeled with either DiIC_16_ (3) (raft-selective dye) or DiIC_12_ (3) (non-raft dye) for 15 min on ice. After labeling, cells were fixed with 3.7% formaldehyde for 10 min, blocked with 5% milk-PBS for 15 min, and permeabilized with 0.5% Triton X-100. Cells were subsequently incubated with a human anti-spike (S) monoclonal antibody (CDC), followed by Alexa Fluor 488-conjugated anti-human IgG secondary antibody. Samples were mounted and imaged by fluorescence microscopy. Methyl-β-cyclodextrin treatment resulted in a marked reduction in spike protein-positive puncta at the membrane and disrupted colocalization with DiIC_16_ (3), supporting the role of cholesterol-rich microdomains in viral particle assembly.

### 2.7. Microarray and qPCR Procedures

To assess host cellular responses induced by SARS-CoV structural protein expression and to explore host pathways involved in lipid raft-associated viral assembly, we performed gene expression profiling using microarray analysis, followed by validation of key targets through quantitative real-time PCR (qPCR). Briefly, total RNA was extracted from mock and S + M + N-transfected HEK-293T cells at 24 h post-transfection using the RNeasy Mini Kit (Qiagen, Hilden, Germany), followed by DNase I treatment to remove genomic DNA contamination. RNA integrity was confirmed using agarose gel electrophoresis. For microarray analysis, radiolabeled cDNA probes synthesized from 5 µg total RNA were hybridized to commercial human gene expression arrays (Atlas Human Cancer Array, Clontech Laboratories, Inc., Mountain View, CA, USA). Differential expression was analyzed by densitometry and validated using quantitative PCR.

For qPCR, 1 µg of total RNA was reverse-transcribed using the SuperScript III First-Strand Synthesis System (Invitrogen, Carlsbad, CA, USA). qPCR was performed using SYBR Green Master Mix (Applied Biosystems, Foster City, CA, USA) on an ABI Prism 7500 system with gene-specific primers. Expression was normalized to 18S rRNA, and relative fold changes were calculated using the ΔΔCt method from three biological replicates.

### 2.8. Image Analysis

Quantitative image analysis was performed using ImageJ/Fiji (ImageJ, version 2.14.0; National Institutes of Health, Bethesda, MD, USA) with uniform thresholding parameters; colocalization was assessed using Manders’ overlap and Pearson correlation coefficients, VLP puncta were counted using predefined size and intensity thresholds, and a minimum of 30 cells per experiment were analyzed across three independent biological replicates.

### 2.9. Statistical Analysis

All experiments were performed independently at least three times. Quantitative data are presented as mean ± SD. Statistical significance was determined using two-tailed unpaired Student’s *t*-tests or one-way ANOVA, as appropriate, and Pearson correlation analysis was used for colocalization studies, with *p* < 0.05 considered statistically significant.

## 3. Results

### 3.1. S, M, and N Proteins Are Required for VLP Budding

**Figure 1:**
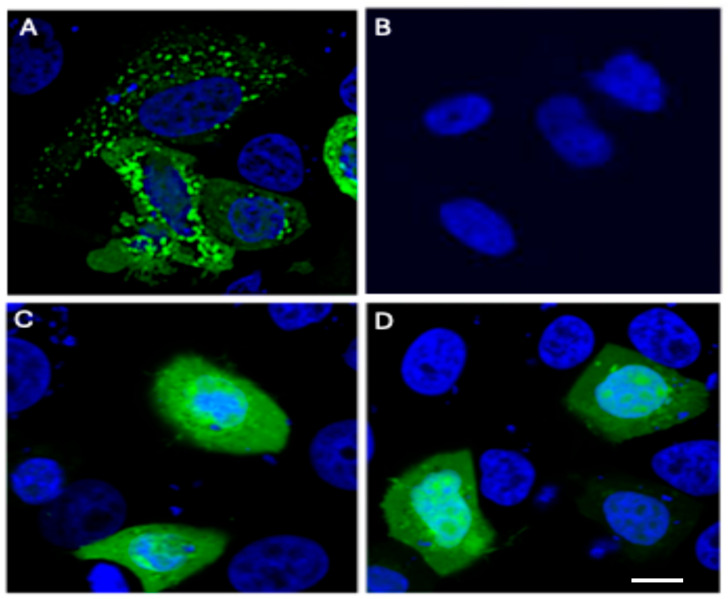
Contribution of different combinations of SARS-CoV S, M and N genes to the formation of Coronavirus-like particles by immunofluorescence. Two microgram of each of the three plasmids encoding S, M, and N were used in different combinations of SMN (**A**), Vector control (**B**), SM (**C**) or SN (**D**) to cotransfect 3 × 10^6^ 293T cells, using calcium phosphate method (6 µg total DNA per transfection). Forty-eight hours after transfection, cells were fixed with 3.7% formaldehyde and stained using human anti-spike anti-bodies followed by Alexa-Fluor 488-conjugated anti-human IgG antibody (green), and nuclei were counter-stained with DAPI (blue). Fluorescent images were obtained with Zeiss Axioplan light microscope at 100× oil immersion. Scale bar = 10 μm. All experiments were performed independently at least three times, unless otherwise stated.

Immunofluorescence revealed that only co-expression of S + M + N yielded discrete, intracellular membrane-associated VLPs (Figure 1A). Approximately 70–80% of S + M + N-transfected cells showed distinct punctate surface staining. In contrast, SM or SN combinations showed diffuse intracellular localization without detectable budding structures at the membrane (Figure 1C,D), confirming that S is essential alongside M and N for VLP envelopment and release, as previously reported for SARS-CoV pseudoparticles [12]. To further confirm these findings at the ultrastructural level, we have included Supplementary Figure S1, adapted from our co-author’s prior work [12], which shows transmission electron microscopy (TEM) images of SARS-CoV-like particles forming in the cytoplasm and budding from intracellular membranes consistent with the ERGIC in S + M + N-transfected 293T cells [12]. The absence of positive-stranded viral genomic RNA, protease, or the viral polymerase indicated that they were not essential for the formation of the SARS-CoV core particles.

### 3.2. VLPs Associate with Lipid Rafts

**Figure 2:**
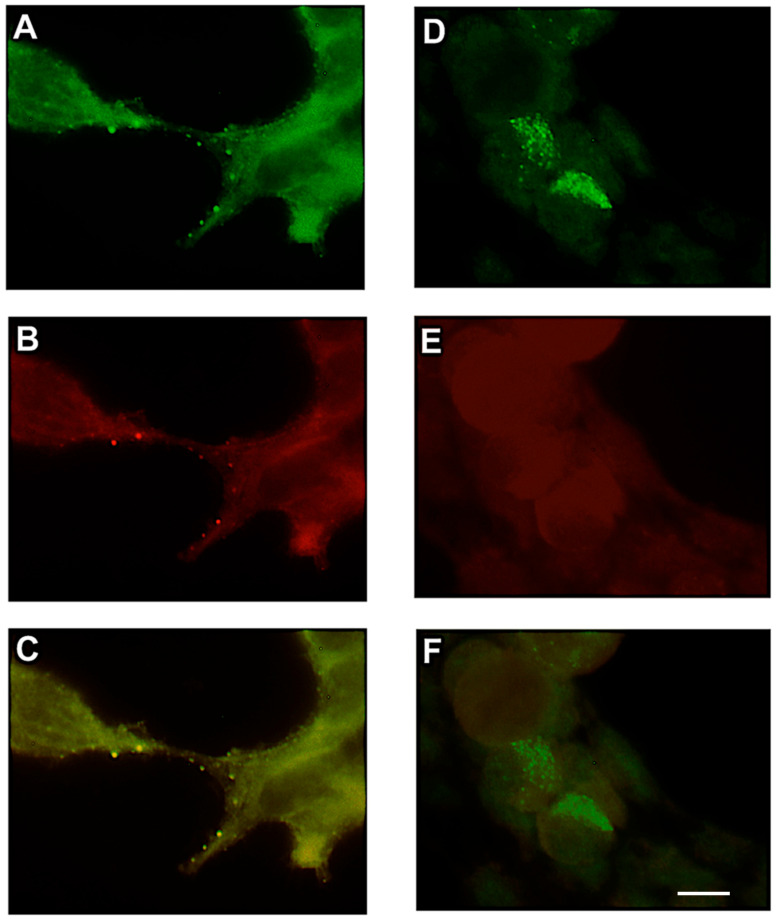
SARS-CoV-like particles colocalize with lipid raft dye DiIC_16_ (3) and not with nonlipid raft dye DiIC_12_ (3). At 48-h post transfection with plasmids encoding S, M and N, cells were incubated with lipid dye DiIC_16_ (3) (**B**) or DiIC _12_ (3) (**E**) for 15 min on ice. Cells were then fixed and labeled with human anti-spike antibody, followed by Alexa-Fluor 488-conjugated anti-human IgG antibody, shown in green (**A**,**D**). Images demonstrate spike glycoprotein colocalizes preferentially with the lipid raft dye DiIC_16_ (**C**) compared to DiIC_12_ (**F**), as demonstrated in yellow. Magnification 100× optovar 2.5. Scale bar = 10 μm. All experiments were performed independently at least three times, unless otherwise stated.

With immunofluorescent markers that selectively partition into liquid-ordered and fluid domains of the membranes, we sought to determine colocalization of viral proteins and cellular proteins with lipid microdomains. At 48 h post-transfection, 293T cells were labeled with either DiIC_16_ (3) (raft-selective dye) or DiIC_12_ (3) (nonraft dye) for 15 min on ice. After fixation, cells were labeled with a human anti-spike (S) monoclonal antibody (CDC), followed by Alexa Fluor 488-conjugated anti-human IgG. DiIC_16_ (raft) staining overlapped strongly with spike-positive particles (Figure 2B,C), while DiIC_12_ (nonraft) showed minimal colocalization (Figure 2E,F). Pearson correlation analysis showed significantly higher co-localization of spike with raft markers (r = 0.82) compared to nonraft dyes (r = 0.14; *p* < 0.0001), supporting the hypothesis that SARS-CoV VLPs preferentially assemble at cholesterol-rich lipid rafts. The puncta observed represent structures on internal membranes consistent with the ERGIC/Golgi region rather than the plasma membrane, as also supported by published TEM studies [12].

#### Spike Protein Localizes to Raft-Enriched Fractions

**Figure 3:**
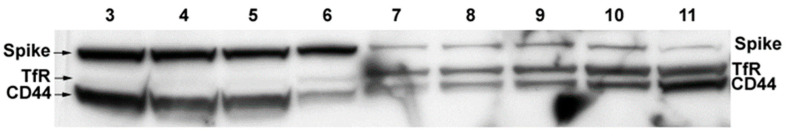
Spike glycoproteins localize to the detergent-insoluble raft microdomains after sucrose gradient separation. Changed to 11293T cells transfected with plasmids encoding S, M, and N were lysed in 1% Triton X-100 at 4 °C at 24 h post-transfection. Postnuclear extract was layered with a discontinuous sucrose gradient and centrifuged at 35,000 rpm for 16 h, and 1-mL fractions were collected. Fraction 1 represents the top of the gradient, 11 represents the bottom, and fractions 3 to 5/6 are where the cholesterol-rich regions of the mem-brane localize. Examination of cellular proteins was performed by Western blotting after separation by sodium dodecyl sulfate-PAGE and transfer to polyvinylidene difluoride membranes. Spike localized to fractions 3 to 5, with the raft protein CD44, while the nonraft protein transferrin receptor (Tfr) was noted in soluble fractions 7 to 11. All experiments were performed independently at least three times, unless otherwise stated.

Identification of proteins associated with the lipid microdomain was performed by cell lysis with the detergent Triton X-100 at 4 °C, followed by sucrose gradient centrifugation as described previously [24]. Selected fractions were then examined for the presence of viral and host cellular proteins by Western blot analysis. Immunoblotting revealed S protein and CD44 within the Triton X-insoluble fractions (fractions 3 to 5), consistent with raft localization (Figure 3), while the nonraft transferrin receptor was found predominantly within the soluble fractions, 7 to 11, validating the specificity of raft partitioning (Figure 3). Although CD44 was enriched in raft fractions, a portion also appeared in heavier fractions, consistent with previous reports that CD44 may partition into both raft and non-raft membranes depending on cell confluence, activation state, and detergent conditions. Therefore, co-fractionation of spike with CD44 in fractions 3–5 supports—but does not alone prove—raft association.

### 3.3. Methyl-β-Cyclodextrin Inhibits Budding

Lipid microdomains are characterized by their high content of cholesterol and sphingolipids. Manipulation of the membranes with cyclic oligosaccharides, such as methyl-β-cyclodextrin, removes cholesterol and disrupts lipid raft formation. To interrogate the functional relevance of rafts in VLP morphogenesis, we disrupted these domains using methyl-β-cyclodextrin. At 48 h post-transfection, 293T cells were treated with 10 mM methyl-β-cyclodextrin (Sigma) for 30 min at 37 °C, washed twice with PBS, and labeled with either the DiIC_16_ (3) or DiIC_12_ (3) dye for 15 min on ice. Cells were fixed with 3.7% formaldehyde, blocked in 5% milk-PBS, and permeabilized with 0.5% Triton X-100. Cells were then labeled with a human anti-spike monoclonal antibody (CDC), followed by Alexa Fluor 488-conjugated anti-human IgG.

**Figure 4:**
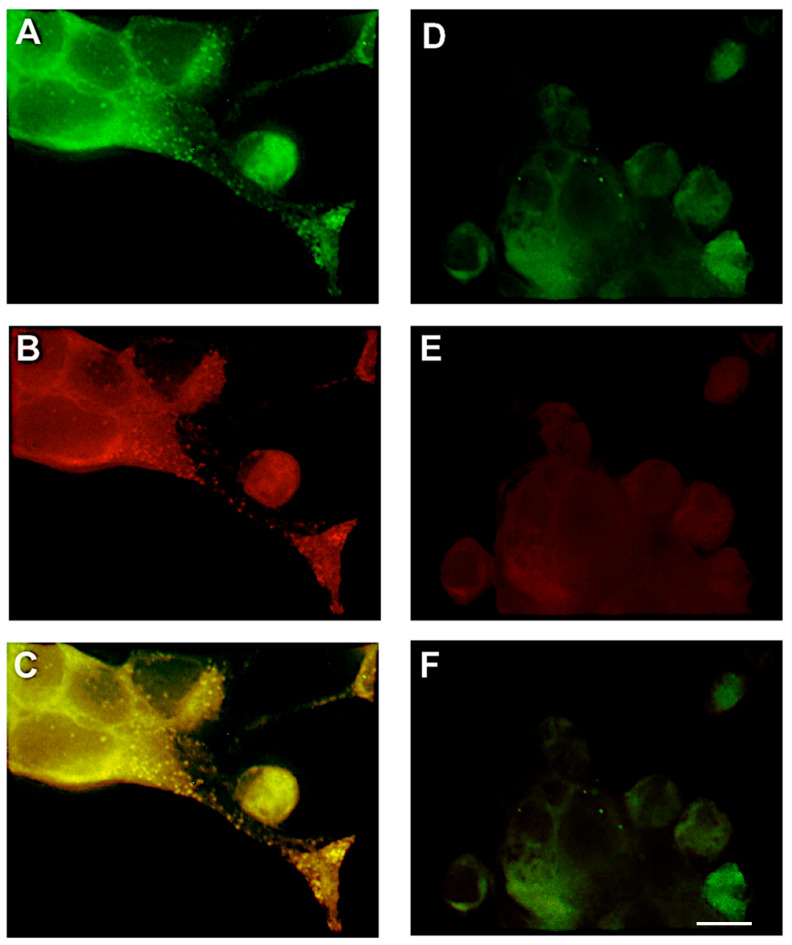
Treatment of 293T cells showing virus-like particles, with methyl-β-cyclodextrin reduces the number of particles within the lipid microdomains. At 48 h post-transfection with plasmids encoding S, M, and N, cells were treated with 10 mM methyl-β-cyclodextrin for 30 min at 37 °C. Staining procedures with lipid dye DiIC16 (3) (**B**,**E**) and with spike antibodies (**A**,**D**) were performed as described in Figure 2 legend. Panel A to C: cells untreated with cyclodextrin showing virus-like particles shown in green (**A**) colocalize with lipid raft dye DiIC16 (3) (**B**) as demonstrated in overlap image (**C**). Panel D to F: cells treated with cyclodextrin showing reduced number of virus-like particles shown in green (**D**) does not colocalize with the lipid raft dye DiIC16 (3) (**E**) as demonstrated in overlap image (**F**). Magnification 100× optovar 2.5. Scale bar = 10 μm. All experiments were performed independently at least three times, unless otherwise stated.

**Figure 5:**
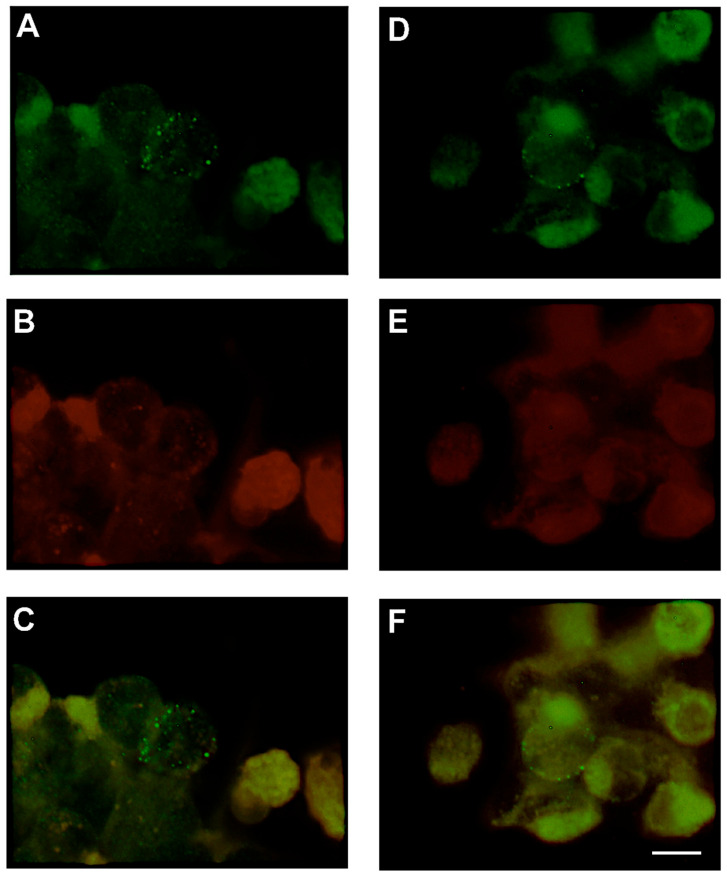
Treatment 293T cells showing virus-like particles, with methyl-β-cyclodextrin does not alter the distribution of particles to nonlipid microdomains. At 48 h post-transfection with plasmids encoding S, M, and N, cells were treated with 10 mM methyl-β-cyclodextrin for 30 min at 37 °C. Staining procedures with nonlipid raft dye DiIC_12_ (3) (**B**,**E**) and with spike antibodies (**A**,**D**) were performed as described in Figure 2 legend. Panel A to C: cells untreated with cyclodextrin showing virus-like particles shown in green (**A**) does not colocalize with nonlipid raft dye DiIC_12_ (3) (**B**) as demonstrated in overlap image (**C**). Panel D to F: cells treated with cyclodextrin showing reduced number of virus-like particles shown in green (**D**) does not colocalize with the nonlipid raft dye DiIC_12_ (3) (**E**) as demonstrated in overlap image (**F**). Magnification 100× optovar 2.5. Scale bar = 10 μm. All experiments were performed independently at least three times, unless otherwise stated.

Cyclodextrin treatment abolished visible VLP budding and disrupted co-localization of spike with DiIC_16_ (3)-labeled lipid raft-like microdomains on internal membranes, including the Golgi apparatus and endoplasmic reticulum (Figure 4D–F). Immunofluorescent image analysis revealed a nearly complete loss of virus-like particles from both the cell surface and internal membranes in cyclodextrin-treated cells (Figure 4D and Figure 5D) compared to untreated controls (Figure 4A–C and Figure 5A–C). A small number of spike-labeled puncta remained in treated cells, but these showed no colocalization with either lipid dye (Figure 4F and Figure 5F). Quantitative image analysis demonstrated >90% reduction in VLP formation (*p* < 0.001). These findings confirm that cholesterol is essential for viral protein organization and particle assembly and suggest that lipid raft disruption may serve as a strategy to impair coronavirus morphogenesis.

#### Quantitative Colocalization Confirms Spike Enrichment in Lipid Rafts

**Figure 6:**
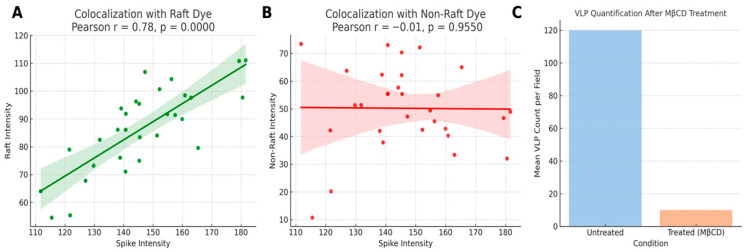
Lipid Raft Colocalization and VLP Inhibition Following Cholesterol Depletion. (**A**) Pearson correlation analysis between SARS-CoV spike protein and raft-associated dye (DiIC16). HEK-293T cells were transfected with plasmids encoding SARS-CoV structural proteins S, M, and N. At 48 h post-transfection, cells were stained with human anti-spike antibody and DiIC16. Scatter plot shows a strong positive correlation (Pearson r = 0.82, *p* < 0.0001), indicating colocalization of spike with cholesterol-rich lipid raft microdomains. Each point in colocalization graphs corresponds to a single cell quantified from ≥3 independent experiments with a minimum of 30 cells analyzed per experiment. (**B**) Pearson correlation analysis between SARS-CoV spike protein and non-raft dye (DiIC12). The same cells were stained with DiIC12, which partitions into non-raft membrane regions. Weak correlation was observed (Pearson r = 0.14, *p* = ns), supporting the specificity of spike localization to lipid rafts. Each point in colocalization graphs corresponds to a single cell quantified from ≥3 independent experiments with a mini-mum of 30 cells analyzed per experiment. (**C**) Quantitative analysis of virus-like particle (VLP) formation before and after cholesterol depletion. HEK-293T cells expressing S, M, and N proteins were treated with 10 mM methyl-β-cyclodextrin (MβCD) to disrupt lipid rafts. Bar graph shows >90% reduction in spike-positive VLPs per microscopic field following treatment, highlighting the importance of cholesterol-rich microdomains in VLP morphogenesis. VLP counts represent individual puncta per microscopic field, averaged from 10 randomly selected fields per experiment. All experiments were performed independently at least three times. Quantitative data are presented as mean ± SD. Statistical significance was determined using two-tailed unpaired Student’s *t*-tests or one-way ANOVA, as appropriate, and Pearson correlation analysis was used for colocalization studies, with *p* < 0.05 considered statistically significant.

Pearson correlation analysis confirmed a strong association between spike protein and raft domains labeled by DiIC16 (Pearson r = 0.82, *p* < 0.0001), while only a weak correlation was observed with DiIC12 (Pearson r = 0.14, ns), indicating specificity for lipid raft microdomains (Figure 6A,B). These data provide quantitative support for spike enrichment within ordered, cholesterol-rich membrane regions.

To further assess the functional relevance of lipid rafts, we quantified spike-positive VLPs before and after cholesterol depletion using methyl-β-cyclodextrin. MβCD treatment reduced VLP formation by >90% compared to untreated controls (*p* < 0.001), as shown by image-based quantification (Figure 6C). This confirms that intact lipid rafts are essential for efficient VLP assembly and membrane localization.

### 3.4. Microarray and qPCR Analysis of Host Gene Expression

**Figure 7:**
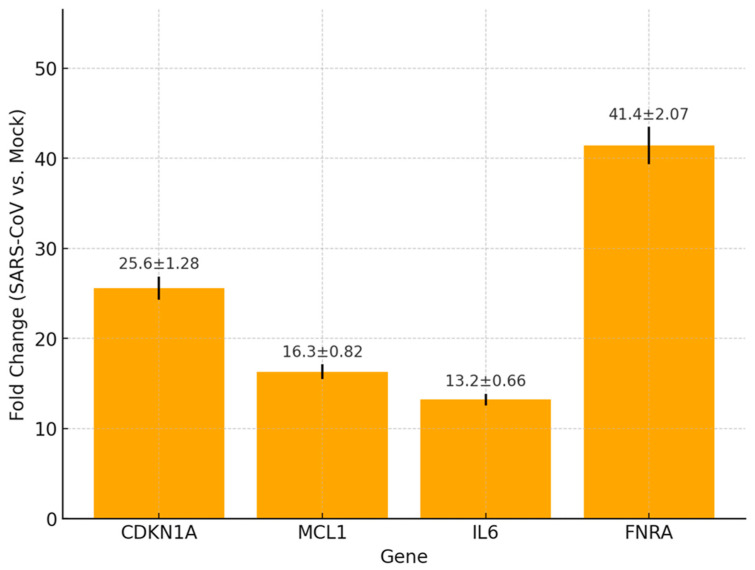
Quantitative real-time PCR (qPCR) validation of host gene expression in HEK-293T cells transfected with SARS-CoV structural proteins (S + M + N) compared to mock-transfected controls. Expression levels of CDKN1A, MCL1, IL6, and FNRA were normalized to 18S rRNA, and fold change was calculated using the ΔΔCt method. Bars represent mean fold change ± standard deviation from three independent experiments performed in triplicate.

To assess host cellular responses induced by SARS-CoV structural protein expression, we performed gene expression profiling using microarray analysis, followed by validation of key targets through quantitative real-time PCR (qPCR) (Figure 7, Table S1 and Supplementary Figure S2).

- Microarray Results

Microarray analysis revealed differential regulation of a subset of host genes in SARS-CoV S + M + N-transfected HEK-293T cells relative to mock controls. Twelve genes were significantly upregulated (≥2-fold, *p* < 0.01), encompassing diverse biological processes including apoptosis (e.g., MCL1, CASP4), stress response (e.g., GADD153), cytoskeletal organization (e.g., VIM, TUBA1), immune modulation (e.g., IL6 (~13-fold)), and cell adhesion (e.g., FNRA). Importantly, several of these upregulated genes—including VIM (vimentin), RHOA (Ras homolog family member A), FNRA (fibronectin receptor α), and CD59—encode proteins that are known to associate with or localize to lipid raft microdomains. These proteins contribute to membrane dynamics, cytoskeletal anchoring, and immune evasion, processes essential for efficient virus assembly and budding. Notably, the most prominently induced transcripts included FNRA (~40-fold), CDKN1A (~25-fold), and MCL1 (~16-fold), all of which are implicated in viral replication dynamics and membrane organization. These results suggest that expression of SARS-CoV structural proteins alone is sufficient to activate transcriptional programs mimicking responses observed during active viral infection, particularly those supporting lipid raft-mediated viral assembly.

b. qPCR Validation

Quantitative real-time PCR confirmed the upregulation of several key genes identified by microarray (Figure 7, Table S1 and Supplementary Figure S2). CDKN1A was upregulated by 25.6-fold, MCL1 by 16.3-fold, IL6 by 13.2-fold, and FNRA by 41.4-fold in transfected cells compared to mock controls. Elevated expression of other raft-associated genes, including VIM, RHOA, and CD59, was also observed, consistent with the formation of a cellular environment conducive to virion assembly within lipid microdomains. These genes are functionally linked to cell cycle regulation, anti-apoptotic signaling, inflammation, cytoskeletal remodeling, and membrane anchoring—biological pathways intersecting with virion budding and egress. The strong concordance between qPCR results and microarray data supports the robustness of the observed transcriptional alterations and underscores the importance of lipid raft–associated gene pathways in facilitating SARS-CoV VLP morphogenesis.

#### Role of Lipid Rafts in SARS-CoV Infection

**Figure 8:**
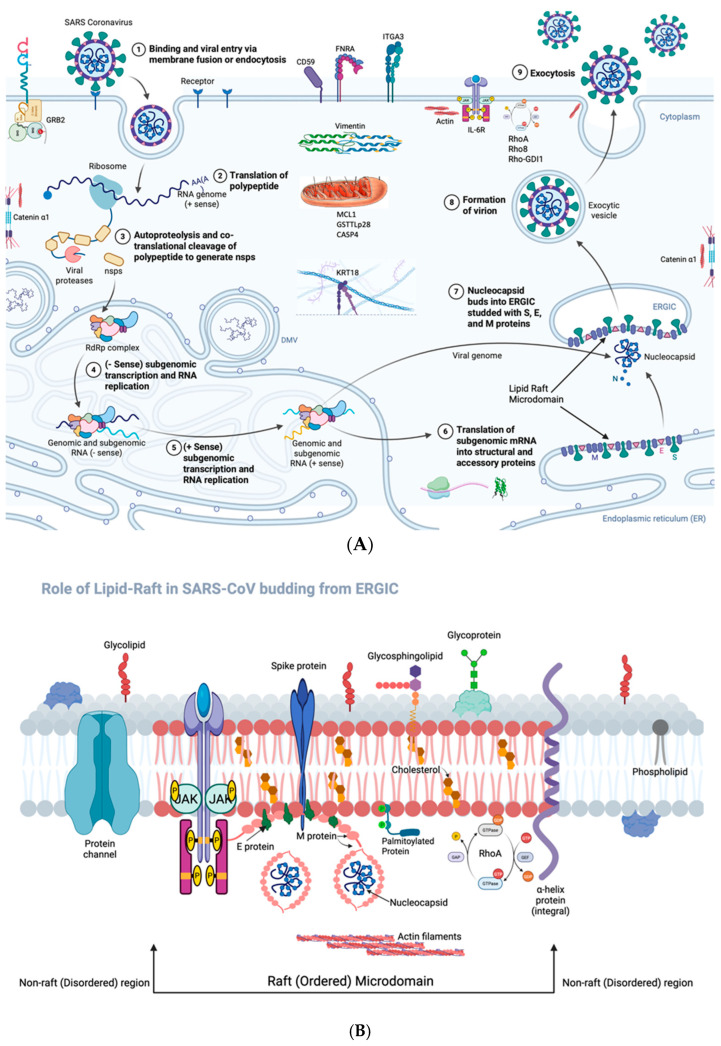
(**A**) Schematic diagram showing role of rafts and host gene expression in SARS-CoV infection. Rafts are involved in virus entry into the host cell and its assembly inside the host cell, as well as release from the plasma membrane. SARS-CoV entry occurs either at rafts in a caveolin- and clathrin-independent manner, or via clathrin-dependent, raft-independent endocytosis. SARS-CoV assembly is thought to involve raft-dependent budding-in of early Golgi membrane (or the membrane of intermediate compartment between the ER and the Golgi--ERGIC), thus enclosing the nucleocapsid. After maturation within the trans-Golgi network (TGN), the enveloped nucleocapsid enters the cytoplasm within a trans-Golgi vesicle through budding-out. Twelve host genes were significantly upregulated (≥2-fold, *p* < 0.01), encompassing diverse biological processes including apoptosis (e.g., MCL1, CASP4), stress response (e.g., GADD153), cytoskeletal organization (e.g., VIM, TUBA1), immune modulation (e.g., IL6), and cell adhesion (e.g., FNRA). Importantly, several of these upregulated genes—including VIM (vimentin), RHOA (Ras homolog family member A), FNRA (fibronectin receptor α), and CD59—encode proteins that are known to associate with or localize to lipid raft microdomains. Created in BioRender. Manoj K. Pastey. (2025). (**B**). Lipid rafts are involved in SARS-CoV assembly. S-palmitoylation of the spike protein facilitates the formation of localized, ordered cholesterol and sphingolipid-rich lipid nanodomains in the early Golgi (or the intermediate compartment between the ER and the Golgi-ERGIC), where viral budding occurs, as well as generating cholesterol-rich lipid domains within viral envelopes [18]. Created in BioRender. Manoj K. Pastey. (2025).

Collectively, the study results are summarized in schematic diagrams (Figure 8A,B), illustrating that lipid rafts facilitate key stages of the SARS-CoV life cycle—including viral entry, assembly, and release—while also highlighting the upregulation of raft-associated host genes that may contribute to viral replication and pathogenesis.

## 4. Discussion

Our findings identify lipid rafts as critical platforms for SARS-CoV-like particle assembly and release. While prior studies have demonstrated the role of lipid rafts in viral entry, our data provide direct experimental evidence that structural SARS-CoV proteins alone are sufficient to induce VLP formation at raft domains, even in the absence of viral genome replication. The requirement for S, M, and N proteins mirrors mechanisms observed in authentic coronavirus infection, wherein S mediates fusion, M drives curvature, and N organizes the viral genome. Although only S protein was stained in this study, future studies using triple immunostaining or fluorescently tagged S, M, and N constructs are warranted to confirm co-expression in individual cells and correlate it with VLP formation. The localization of VLPs to DiIC_16_ (3)-stained domains and biochemical co-fractionation with CD44 confirm that SARS-CoV targets cholesterol-rich microdomains for budding. Although CD44 was enriched in raft fractions, a portion also appeared in heavier fractions, consistent with previous reports that CD44 may partition into both raft and non-raft membranes depending on cell confluence, activation state, and detergent conditions. Therefore, co-fractionation of spike with CD44 in fractions 3–5 supports—but does not alone prove—raft association. While our sucrose gradient Western blot analysis confirmed spike protein localization to detergent-resistant lipid raft fractions, inclusion of membrane (M) and nucleocapsid (N) proteins in future fractionation blots will be essential to fully characterize the composition of raft-associated VLPs and confirm the co-localization of all three structural proteins within these microdomains. Although the co-expression of S, M, and N proteins was sufficient to drive VLP formation in our system based on immunofluorescence and biochemical fractionation, the inclusion of a transmission electron microscopy (TEM) image previously reported [12] (Supplementary Figure S1) offers ultrastructural confirmation of VLP formation and further supports our localization model. The puncta observed represent structures on internal membranes consistent with the ERGIC/Golgi region rather than the plasma membrane, as also supported by published TEM studies [12]. Further, we acknowledge that the envelope (E) protein plays an important role in coronavirus assembly, budding, and virion stability. Previous studies have shown that E protein can influence membrane curvature and scission, potentially enhancing the efficiency of particle release. Inclusion of the E protein in future iterations of our VLP system may improve particle yield, structural fidelity, and possibly the incorporation of additional viral or host factors relevant to assembly. Therefore, planned follow-up experiments will incorporate E protein to determine its impact on VLP morphogenesis and to more closely replicate the composition of authentic SARS-CoV virions.

Lipid rafts are now understood as dynamic nanoscale assemblies of cholesterol and sphingolipids that can coalesce into larger ordered platforms, particularly upon protein clustering or signaling activation [1,8]. Lipid rafts are not merely confined to the plasma membrane. In fact, as reported by numerous studies, lipid microdomains are formed similarly in the subcellular organelles, such as Golgi, ER, endosomes, lysosomes, lipid droplets, mitochondria and nuclei, termed as raft-like microdomains, where they are involved in cargo sorting and vesicle trafficking [25,26,27]. Moreover, host lipid rafts have been reported to be critically involved in apical targeting, assembly, and virus budding. In this case, the subcellular distribution of lipid raft on internal membranes, including the Golgi apparatus or the ER, has a significant impact in the sorting of proteins and in the trafficking and overall exocytosis of viral proteins, which constitute fundamental steps to support viral infection [28,29]. Although the internal membrane localization is consistent with ERGIC involvement, definitive identification of Golgi or ER was not performed in this study and requires co-staining with organelle-specific markers.

Rafts contribute to multiple stages of the viral life cycle, including entry, intracellular assembly, and release. SARS-CoV-2 enters human cells by binding of virus spike glycoprotein (S) to its receptor, angiotensin-converting enzyme 2 (ACE2) in the plasma membrane of the host cell; this entry occurs either at rafts in a caveolin- and clathrin-independent manner, or via clathrin-dependent, raft-independent endocytosis [14,15,16,17,30]. SARS-CoV-2 assembly is thought to involve raft dependent budding-in of early Golgi membrane (or the membrane of intermediate compartment between the ER and the Golgi-ERGIC), thus enclosing the nucleocapsid [30]. After maturation within the trans-Golgi network (TGN), the enveloped nucleocapsid enters the cytoplasm within a trans-Golgi vesicle through budding-out [30].

Our findings reinforce these observations by showing that raft disruption using methyl-β-cyclodextrin significantly impairs VLP budding, suggesting that membrane order and cholesterol content are required for proper protein clustering and particle release (Figure 4, Figure 5 and Figure 6). Importantly, methyl-β-cyclodextrin disrupts these lipid microdomains by extracting cholesterol, ceramides, and fatty acids, and by depolymerizing actin filaments—thus affecting both membrane structure and cellular architecture [8]. Consistent with this, our data show that cyclodextrin treatment markedly reduces (>90% reduction) VLP formation and spike protein colocalization with raft markers. While these findings strongly suggest that cholesterol-rich microdomains are required for proper structural protein clustering but do not yet establish that VLP budding itself strictly depends on raft integrity, and further biochemical validation is therefore warranted. In particular, Western blot analysis of VLPs purified from culture supernatants following methyl-β-cyclodextrin treatment would provide quantitative evidence for the reduction in structural protein incorporation and particle release. Incorporating this approach in future experiments will allow us to directly compare VLP yields between treated and untreated conditions and further substantiate the critical role of cholesterol-rich microdomains in coronavirus morphogenesis.

The disruption of VLP release by methyl-β-cyclodextrin reinforces the importance of raft integrity. Similar results have been observed with RSV and HIV, suggesting a conserved mechanism among enveloped viruses [4,6]. Recent studies indicate that SARS-CoV-2 also requires intact rafts for ACE2 clustering and entry, and that lipid-altering drugs like statins can reduce infectivity by modulating raft composition [9,15].

Our transcriptomic analysis further highlights this dependence. Microarray and qPCR validation revealed significant upregulation of raft-associated host genes such as FNRA, VIM, CD59, and RHOA (Figure 7, Table S1; Supplementary Figure S2). Notably, the most prominently induced transcripts included FNRA (~40-fold), CDKN1A (~25-fold), and MCL1 (~16-fold), all of which are implicated in viral replication dynamics and membrane organization. These genes are involved in cytoskeletal anchoring, membrane remodeling, and immune evasion—processes that collectively support virion morphogenesis. The transcriptional induction of these genes by S + M + N expression alone suggests that SARS-CoV structural proteins can reprogram host cell pathways to promote raft-dependent assembly. While these findings reflect host transcriptional responses to SARS-CoV structural protein expression but do not demonstrate functional roles in VLP assembly; therefore, their specific functional contributions remain to be determined. Future studies will employ targeted approaches such as siRNA-mediated knockdown, CRISPR-Cas9 gene editing, or dominant-negative mutants to disrupt individual gene expression, followed by rescue experiments to assess the impact on VLP assembly and release. These mechanistic investigations will be critical to confirm whether the observed transcriptional changes directly contribute to lipid raft organization, cytoskeletal remodeling, or membrane curvature during coronavirus morphogenesis.

VLPs are emerging as powerful vaccine platforms, offering safety advantages by lacking viral genomes while preserving native virion structure. While our findings provide preliminary evidence for raft-dependent assembly of SARS-CoV VLPs, further quantitative validation and comparison with advanced VLP platforms, such as those described previously [31], will be essential to evaluate their utility for translational applications. Moreover, raft-localized VLPs may inherently enhance immunogenicity through interaction with immune receptors embedded in lipid microdomains.

Collectively, these data expand our understanding of coronavirus-host interactions by demonstrating that SARS-CoV VLPs exploit lipid rafts not only for spatial organization but also for inducing supportive host responses (Figure 8A,B). Our findings align with studies on other enveloped viruses, indicating a conserved mechanism for lipid raft utilization. The selective localization of SARS-CoV structural proteins to raft domains and the dramatic inhibition of VLP budding upon raft disruption suggest that targeting lipid raft pathways could offer promising antiviral strategies. Furthermore, our data support lipid raft-associated proteins’ roles in modulating cellular responses conducive to viral replication and assembly, emphasizing their significance as potential therapeutic targets.

## 5. Conclusions

SARS-CoV S, M, and N proteins are sufficient for VLP production, which occurs preferentially at lipid raft microdomains. Disruption of rafts impairs this process, highlighting cholesterol-rich membranes as essential for coronavirus assembly. This study provides a critical foundation for developing raft-targeted antiviral therapies and designing effective VLP-based coronavirus vaccines.

## Data Availability

The original contributions presented in this study are included in the article/Supplementary Materials. Further inquiries can be directed to the corresponding author.

## Supplementary Materials

The following supporting information can be downloaded at: https://www.mdpi.com/article/10.3390/microorganisms14010159/s1, Figure S1. Formation of coronavirus-like particles by inclusion of S glycoprotein expression vector. Electron micrograph of virus particles in HEK-293T cells transfected by the calcium phosphate method (total of 8 μg of DNA per transfection), with plasmids encoding SARS-CoV S, M, and N proteins. Panel (A) shows a high-magnification view of VLPs forming in the cytoplasm adjacent to the nuclear membrane (×30,000). Panel (B) shows a VLP with a corona-like structure emerging from an intracellular membrane (×200,000). (Adapted with permission from co-author [12]; doi: 10.1128/JVI.78.22.12557-12565); Figure S2. Comparison of microarray and quantitative real-time PCR (qPCR) fold-change values for host genes significantly upregulated in HEK-293T cells co-transfected with SARS-CoV S, M, and N structural protein expression vectors. Bars represent mean fold-change ± standard deviation (SD) from three independent experiments (*p* < 0.01). Microarray data (yellow) and qPCR data (orange) show strong concordance, with key raft-associated genes—VIM, RHOA, FNRA, and CD59—exhibiting marked induction. These genes encode proteins involved in membrane dynamics, cytoskeletal anchoring, and immune modulation, consistent with lipid raft–mediated viral assembly; Table S1. Microarray and quantitative real-time PCR (qPCR) fold-change values for significantly upregulated host genes in HEK-293T cells co-transfected with SARS-CoV S, M, and N structural protein expression vectors. Fold-change values are expressed relative to mock-transfected controls. Microarray analysis identified genes with ≥2-fold upregulation (*p* < 0.01), encompassing functional categories such as apoptosis (MCL1, CASP4), stress response (GADD153), cytoskeletal organization (VIM, TUBA1), immune modulation (IL6), and cell adhesion (FNRA). Several upregulated genes (VIM, RHOA, FNRA, CD59) are known to localize to lipid raft microdomains, implicating them in virus assembly and budding processes. qPCR validation confirmed the transcriptional changes observed in the microarray, demonstrating strong concordance between both methods.
