# Supplementary material for: Selective Budding of SARS-CoV-Like Particles from Glycolipid-Enriched Membrane Lipid Rafts and Host Gene Modulation
Source: Microorganisms. 2026 Jan 10;14(1):159. doi: 10.3390/microorganisms14010159 (PMC12844276; doi:10.3390/microorganisms14010159)
Supplement: Supplementary file 1 [file microorganisms-14-00159-s001.zip › microorganisms-4011026-supplementary.pdf]

Supplementary Materials

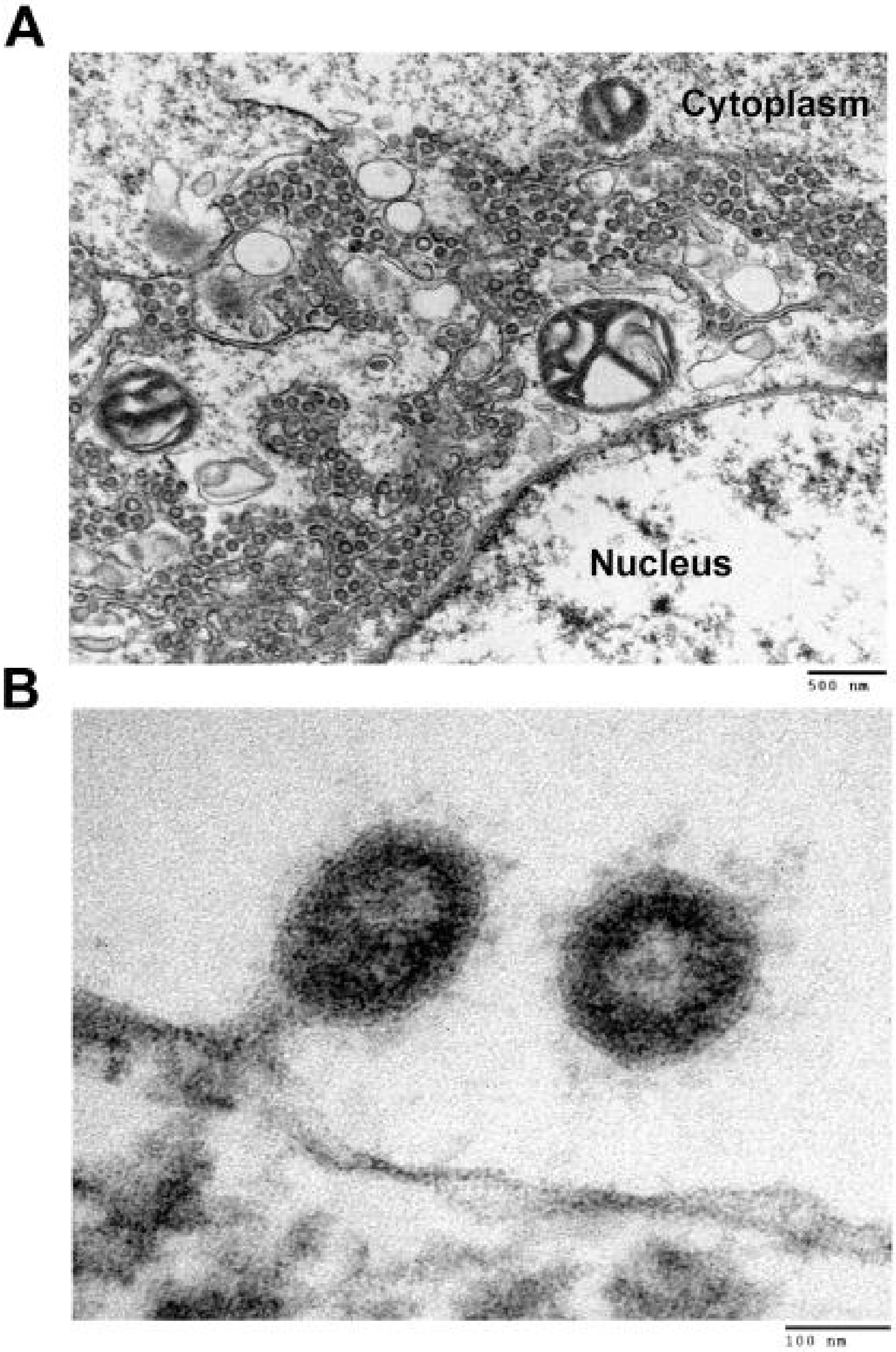

Supplementary Figure S1. Formation of coronavirus-like particles by inclusion of S glycoprotein expression vector. Electron micrograph of virus particles in HEK-293T cells transfected by the calcium phosphate method (total of 8 μg of DNA per transfection), with plasmids encoding SARS-CoV S, M, and N proteins. Panel (A) shows a high-magnification view of VLPs forming in the cytoplasm adjacent to the nuclear membrane (×30,000). Panel (B) shows a VLP with a corona-like structure emerging from an intracellular membrane (×200,000). (Adapted with permission from co-author Huang Y et al., 2004; doi: 10.1128/JVI.78.22.12557-12565).

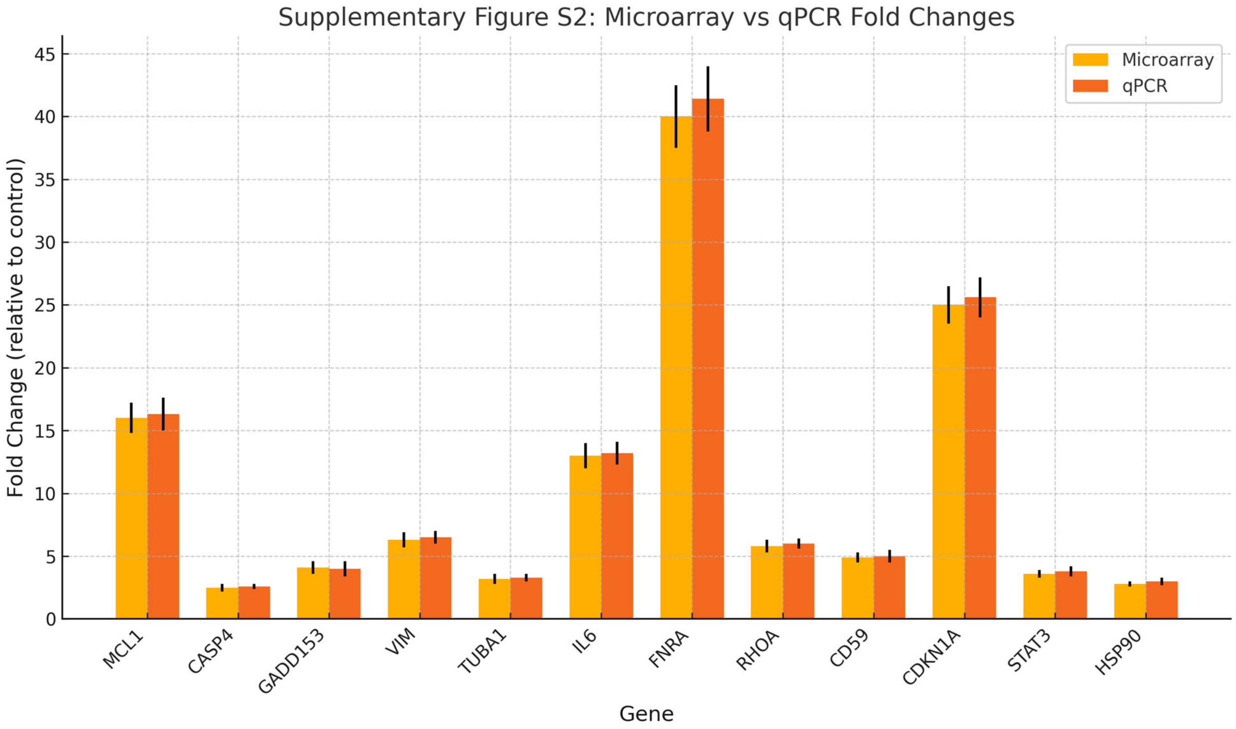

Supplementary Figure S2: Comparison of microarray and quantitative real-time PCR (qPCR) fold-change values for host genes significantly upregulated in HEK-293T cells co-transfected with SARS-CoV S, M, and N structural protein expression vectors. Bars represent mean fold-change ± standard deviation (SD) from three independent experiments ($p < 0.01$). Microarray data (yellow) and qPCR data (orange) show strong concordance, with key raft-associated genes—VIM, RHOA, FNRA, and CD59—exhibiting marked induction. These genes encode proteins involved in membrane dynamics, cytoskeletal anchoring, and immune modulation, consistent with lipid raft–mediated viral assembly

Supplementary Table S1: Microarray and quantitative real-time PCR (qPCR) fold-change values for significantly upregulated host genes in HEK-293T cells co-transfected with SARS-CoV S, M, and N structural protein expression vectors. Fold-change values are expressed relative to mock-transfected controls. Microarray analysis identified genes with ≥2-fold upregulation ($p < 0.01$), encompassing functional categories such as apoptosis (MCL1, CASP4), stress response (GADD153), cytoskeletal organization (VIM, TUBA1), immune modulation (IL6), and cell adhesion (FNRA). Several upregulated genes (VIM, RHOA, FNRA, CD59) are known to localize to lipid raft microdomains, implicating them in virus assembly and budding processes. qPCR validation confirmed the transcriptional changes observed in the microarray, demonstrating strong concordance between both methods.

Gene	Microarray_FC	qPCR_FC
FNRA	40.0	41.4
CDKN1A	25.0	25.6
MCL1	16.0	16.3
IL6	13.0	13.2
VIM	5.0	5.2
RHOA	4.5	4.7
CD59	3.5	3.6
CASP4	3.2	3.4
GADD153	3.0	3.1
TUBA1	2.8	2.9
Gene11	2.5	2.6
Gene12	2.3	2.4
